# Antifungal Efficacy of a Thiazolyl Hydrazone Derivative Incorporated into a Self-Emulsifying Drug Delivery System in a Murine Model of Cryptococcosis

**DOI:** 10.1021/acsomega.5c02705

**Published:** 2025-06-21

**Authors:** Iara R. Silva, Victor A. T. Leocádio, Gustavo J. C. Freitas, Renata B. Oliveira, Elaine A. Leite, Daniel A. Santos, Isabela C. César

**Affiliations:** † Departamento de Produtos Farmacêuticos, Faculdade de Farmácia, 28114 Universidade Federal de Minas Gerais, Av. Antônio Carlos, 6627, Pampulha, Belo Horizonte, Minas Gerais 31270-901, Brazil; ‡ Departamento de Microbiologia, Instituto de Ciências Biológicas, Universidade Federal de Minas Gerais, Av. Antônio Carlos, 6627, Pampulha, Belo Horizonte, Minas Gerais 31270-901, Brazil; § National Institute of Science and Technology in Human Pathogenic Fungi, Av. Antônio Carlos, 6627, Pampulha, Belo Horizonte, Minas Gerais 31270-901, Brazil

## Abstract

2-[2-(Cyclohexylmethylene)­hydrazinyl]-4-phenylthiazole (RN104) has shown notable antifungal activity against *Cryptococcus* species and low toxicity. This study aimed to assess the antifungal efficacy and survival benefits of RN104 incorporated into a self-emulsifying drug delivery system (SEDDS-RN104) compared to free RN104 and fluconazole (FCZ) in a murine model of *Cryptococcus neoformans* (H99) infection. The results demonstrated that SEDDS-RN104 significantly enhanced survival rates compared to free RN104 and FCZ, reducing the event rate (death) by 78% compared to FCZ. The mean survival time was 25 days in the SEDDS-RN104 group, compared to 14 days for free RN104 and 19 days for FCZ. Additionally, the fungal burden in the lungs was markedly reduced in the SEDDS-RN104 group, as confirmed by histopathological analysis. These findings suggest that SEDDS-RN104 effectively addresses the pharmacokinetic limitations of RN104, enhancing its antifungal efficacy and positioning it as a promising therapeutic alternative for cryptococcal infections.

## Graphical abstract

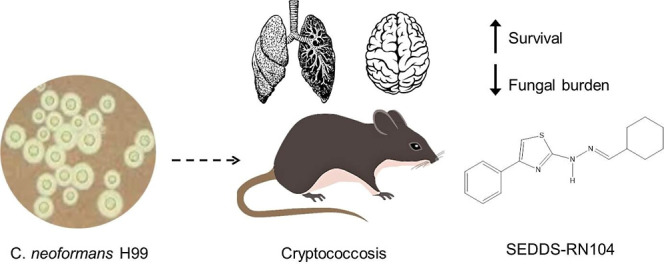

## Introduction

Cryptococcosis is a severe infection affecting both immunocompromised and immunocompetent patients.^1^ While the *Cryptococcus* fungus is primarily known for causing meningoencephalitis, it can also infect various other organs in the body. Pulmonary infection is the second most common manifestation of the disease.^2^ Two yeast species, *Cryptococcus neoformans* and *Cryptococcus gattii*, are the main etiological agents of this disease. As a neglected disease, particularly in regions with limited healthcare resources, cryptococcosis often receives less attention and investment for treatment compared to other infectious.^3,4^ *Cryptococcus* spp. is also major contributors to invasive fungal infections, posing a significant threat to human health.^5^

Current treatments, which include amphotericin B and flucytosine or fluconazole (FCZ), are limited by host toxicity, particularly with amphotericin B and flucytosine, and by the emergence of resistance, notably with FCZ.^6^ Additionally, neurocryptococcosis presents specific therapeutic challenges that are particularly pronounced in resource-limited settings. Effective disease management requires prolonged treatment with antifungals, such as amphotericin B and flucytosine, which are often unavailable or unaffordable in many low- and middle-income countries. While FCZ, although is more accessible and less toxic, its fungistatic nature and the growing incidence of resistant strains contribute to treatment failure. These limitations highlight the urgent need for safer, orally administered, and more effective therapeutic alternatives.^7,8^

In this context, our research group developed a series of thiazolyl hydrazone derivatives, among which 2-[2-(cyclohexylmethylene)­hydrazinyl)]-4-phenylthiazole (RN104) (Figure 1) stands out for its relevant activity against the *Cryptococcus* genus. It demonstrated potent antifungal activity against *C. neoformans* and *C. gattii*, with a minimum inhibitory concentration (MIC) of 0.9 μM for both fungi, surpassing the activity of FCZ used as a positive control (MIC = 3.2 μM for *C. neoformans* and 7.8 μM for *C. gattii*).^9^

**Figure 1:**
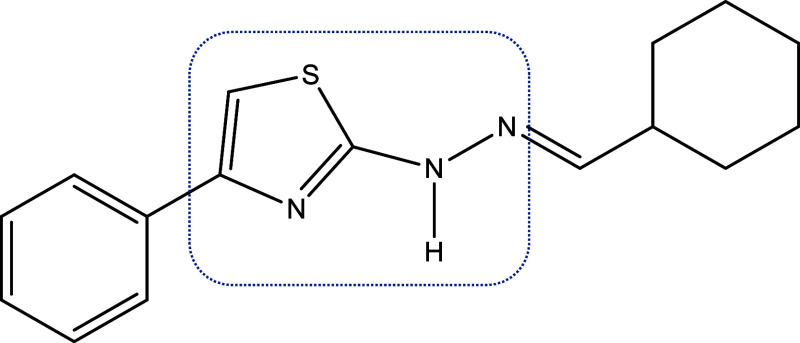
RN104 chemical structure with the thiazolyl hydrazone moiety highlighted in blue.

The preclinical toxicity evaluation revealed a low cytotoxic profile in the tested cell lines, including human lung (A549), rat heart (H9C2), human liver (HepG2), porcine kidney (LLC-PK1), and mouse brain (NEURO-2), and in vivo acute toxicity study was also conducted.^10^ The effect of RN104 on *Cryptococcus* spp. has been previously demonstrated in vitro and in vivo, leading to increased intracellular oxidative stress.^11^ Additionally, this drug may influence the fungal virulence, causing a decreased biofilm formation and capsule thickness.^12^

The high lipophilicity (log *P* = 5.84) and, consequently, the low aqueous solubility of RN104 negatively affect its oral bioavailability due to reduced dissolution and absorption. To enhance the compound’s aqueous solubility, an inclusion complex was proposed using RN104 and different cyclodextrins: β-cyclodextrin (β-CD), 2-hydroxypropyl-β-CD (2-HP-β-CD), and γ-CD. Using the kneading technique, inclusion in 2-HP-β-CD at a 1:1 molar ratio resulted in the greatest solubility of RN104 (25.88%). However, the increase in aqueous solubility achieved through complexation with CDs did not significantly impact the oral bioavailability of RN104.^13,14^

The preclinical pharmacokinetic study in mice demonstrated that the developed self-emulsifying drug delivery system (SEDDS) formulation effectively enhanced RN104 release in physiological environments, successfully overcoming its limited solubility in aqueous media. Additionally, the formulation presented a prolonged *T*_max_ and *T*_1/2_, a 3-fold reduction in elimination rate constant (*K*_el_), and decreased clearance, suggesting a sustained release profile and reduced metabolism, leading to significant pharmacokinetic improvements. The bioavailability of SEDDS-RN104 increased approximately 21 times compared to that of free RN104. These data indicate that the formulation dramatically enhanced systemic exposure. Another notable aspect of SEDDS-RN104 involves its potential for absorption via the lymphatic system, thus bypassing hepatic first-pass metabolism.^15^ These advancements underscore the potential of the SEDDS formulation to establish RN104 as a promising antifungal candidate.

Thus, this study aimed to evaluate the antifungal efficacy of the SEDDS-RN104 formulation in a murine model of *Cryptococcus* infection.

## Materials and Methods

### Antimicrobial Agents and Reagents

RN104 was synthesized in-house at the Laboratório de Química Farmacêutica of Universidade Federal de Minas Gerais (Belo Horizonte, MG, Brazil) following the previously developed method.^9^ FCZ (Sigma-Aldrich) was commercially acquired and used as a positive control. Medium chain triglyceride (MCT) was kindly provided by Lipoid GMbH (Ludwigshafen, Germany). Super refined polysorbate 80 (Tween 80) was kindly donated by Croda Health Care (Snaith, UK). Sorbitan monooleate (Span 80), sodium carboxymethyl cellulose, and Sabouraud dextrose agar (SDA) were purchased from Sigma-Aldrich (Saint Louis, USA). Ultrapure water was obtained from a Millipore system (Bedford, MA, USA).

### SEDDS-RN104 Formulation

The SEDDS-RN104 formulation consisted of an isotropic mixture of MCT, polysorbate 80, and sorbitan monooleate in a ratio of 65.5:23:11.5 (w/w), with 0.1% (w/w) ascorbyl palmitate added as an antioxidant. It forms a nanometer-scale oil-in-water emulsion in situ in the gastrointestinal tract upon oral administration. SEDDS-RN104 achieved maximum drug loading (10 mg/mL) and a particle size of 118.4 ± 0.7 nm.^15^

### In Vivo Studies

#### Ethics

This study was approved by the Ethics Committee in the Use of Animals (CEUA) of the Universidade Federal de Minas Gerais (protocol 79/2024). We followed the Brazilian Society of Zootechnics/Brazilian College of Animal Experimentation guidelines and Federal Law 11.794/2008. Water and food were provided ad libitum, and light/dark cycles were maintained. Only healthy animals, 6 weeks old and weighing approximately 23 g, were included in the study. All efforts to minimize the suffering of the animals were carried out.

### Evaluation of Survival in a Murine Model of *C. neoformans* Infection

For infection, C57/BL6 female mice (20–23 g) were anesthetized with ketamine and xylazine (80 and 15 mg/kg, respectively). Intratracheal infection was carried out by a small incision in the skin, close to the thyroid, and after separating the tissue layers, the trachea was exposed and inoculated with 1 × 10^5^ cells of *C. neoformans* H99 in 30 μL, and then the incision was sutured. *C. neoformans* H99 was previously cultivated in SDA at 35 °C for 48 h. The cells were then transferred to sterile saline solution (0.9% NaCl), and the inoculum was counted using a Neubauer chamber with Trypan blue staining and standardized.^17^

Mice were divided into groups (*n* = 6) according to the treatments and administration routes^17^ (intraperitoneally (10 mg/kg) and per os (50 mg/kg) once daily), starting 24 h after infection: (1) RN104 free, (2) SEDDS-RN104, (3) FCZ, (4) SEDDS blank, (5) untreated, and (6) noninfected (NI). The RN104 doses were based on previous studies conducted with this drug.^12–15^ Mice were monitored daily, and animals showing weight loss greater than 20%, tremors, or immobility were euthanized in accordance with CONCEA standards (National Council for Control of Animal Experimentation).

### Determination of Fungal Burden

Following the survival evaluation, additional tests were performed to assess the fungal load after infection and per os treatment. The infection and treatment protocols were conducted as previously described at the same doses. 10 days postinfection (dpi), the animals were euthanized (*n* = 8/group), and their lungs and brains were harvested to evaluate fungal burden. By 10 days postinfection, the fungal burden is typically well established in key target organs, allowing for clear visualization of tissue colonization and pathological changes. Additionally, this time point permits the evaluation of treatment efficacy before the onset of extensive tissue damage or mortality.^17^ After homogenizing the organs in phosphate buffer solution, 50 μL of each lung and 200 μL of each brain were plated on SDA and incubated at 37 °C for 48 h. Subsequently, colonies were visually counted, and the number of CFU/g of each organ was determined.

### Histological Analysis

The lungs were collected (*n* = 2/group) and preserved in 10% formalin solution for 24 h. After this period, the organs were transferred to 70% ethanol and kept in this solution for 24 h. Each organ was then sectioned into segments and placed in a histological cassette for slide preparation. The lung tissues were stained with hematoxylin–eosin. Tissue visualization was performed using an optical microscope (amplification: 100×), and the respective images were captured. The number of yeast cells and the visual appearance of the tissue were analyzed and classified using a scoring system from 0 to 5.^18^

### Statistical Analysis

Statistical analyses were performed using GraphPad Prism version 9.5 (GraphPad Inc., San Diego, CA, USA), with *p* < 0.05 considered to be significant. The survival curve was plotted using the Kaplan–Meier method, and the results were analyzed using the *log-rank* test and hazard ratio (HR). CFU and histological scores were analyzed for significant differences using one-way analysis of variance at a 95% significance level, followed by Tukey’s test.

## Results and Discussion

### Lethality Is Reduced in Mice Treated with SEDDS-RN104

We evaluated the effects of RN104 on murine cryptococcosis. It can be observed in Figure 2A that the survival curve following oral treatment with SEDDS-RN104 was above all others, suggesting that animals receiving the developed formulation have a higher probability of surviving longer than those receiving FCZ or free RN104. Figure 2B shows that animals treated with FCZ and SEDDS-RN104 administered intraperitoneally displayed closely aligned curves from day 30 onward.

**Figure 2:**
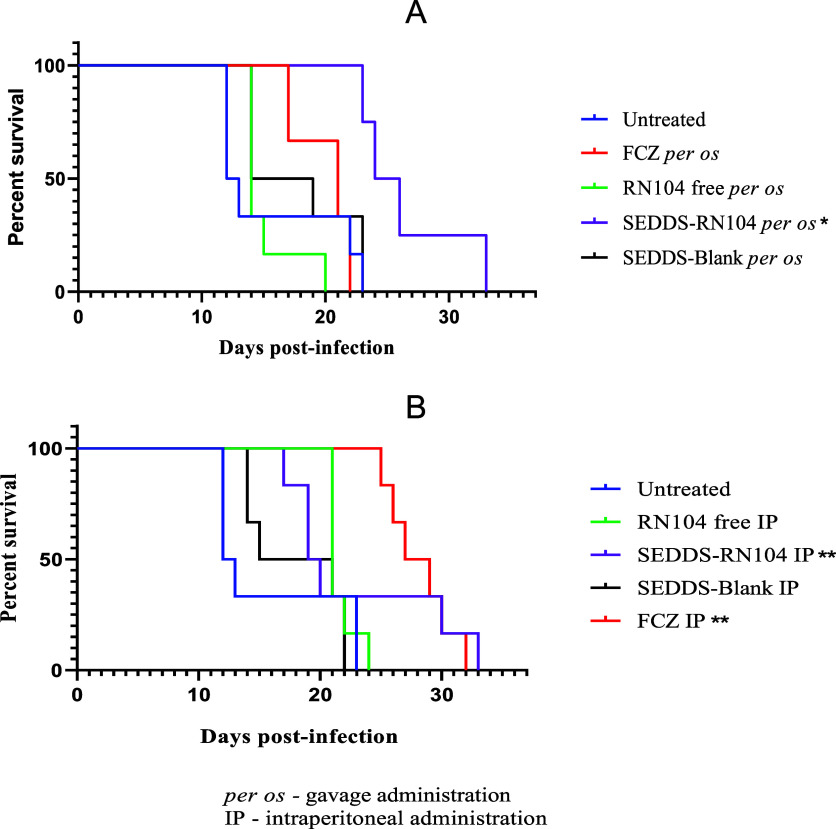
Kaplan–Meier curves for treatments administered by per os (A) and intraperitoneal (B) routes; * (*p* < 0.05 in relation to the untreated group and the other treatments); ** (*p* < 0.05 in relation to the untreated group).

As an initial step in the statistical analysis, the median survival time in dpi was estimated for each treatment based on the curves (Table 1). As expected, the shortest lifespan was recorded in the infected, untreated group, with the first death occurring on 12th dpi. The impact analysis of various treatments administered per os on lethality revealed that SEDDS-RN104 significantly delayed the onset of mortality, with the first animal succumbing on the 23rd dpi. The longest survival time was observed with the intraperitoneal administration of FCZ, with the first death occurring on 25th dpi.

**Table 1:** Survival Times (dpi) of Animals Subjected to Different Treatments^a^

treatments	median (dpi)	first death (dpi)
untreated	12.5	12
RN104 free per os	14	14
SEDDS-RN104 per os	25	23
SEDDS-blank per os	16.5	14
FCZ per os	19	17
RN104 free IP	21	22
SEDDS-RN104 IP	20	17
SEDDS-blank IP	17	14
FCZ IP	28	25

a d.p.i = days postinfection; IP = intraperitoneal; FCZ = fluconazole.

To compare the treatments, results were grouped by routes of administration, and the log-rank test was applied. Under the hypothesis of equality of survival curves, this test yielded a chi-square (χ^2^) value of 13.04 for the per os route and 13.81 for the intraperitoneal route, resulting in *p*-values of 0.0079 and 0.0111, respectively, indicating a significant difference between the treatments (*p* < 0.05).

To identify which treatments differed within the same route of administration, pairwise comparisons were performed by using the log-rank test combined with the assessment of the HR. The HR is calculated as the ratio of the slopes of the survival curves, a measure of the rate at which individuals in a group die.^16^

When comparing FCZ and SEDDS-RN104 administered per os, a *p*-value of 0.0067 was obtained from the log-rank test, indicating a significant difference between these treatments (*p* < 0.05), with a HR (SEDDS-RN104/FCZ) of 0.22. This HR value reflects a 78% reduction in the event rate (death) when administering SEDDS-RN104 versus FCZ. The formulation also demonstrated significant superiority over RN104 administered in its free form (*p* = 0.0024; HR SEDDS-RN104/RN104 free = 0.27).

Additionally, when analyzing the data from the untreated group compared to the group treated with the blank formulation (SEDDS-Blank) per os, there was no statistical evidence of a difference between them (χ^2^ = 1.33; *p* = 0.2485). This fact suggests that the developed formulation is not toxic to animals at the daily administered dose. Another point reinforcing this conclusion is that the variation in body weight between these groups did not show significant differences (*p* = 0.9999), indicating that the formulation does not adversely affect the animals’ weight (Figure 3A). The weight loss in the untreated group (−22.71 ± 2.16%) was similar to that in the SEDDS-Blank group (−22.58 ± 3.36%), suggesting that it is likely related to the course of the disease. Notably, the least weight loss was observed in the group treated with SEDDS-RN104 per os (−1.84 ± 6.32), which was significantly different from the other treatment groups.

**Figure 3:**
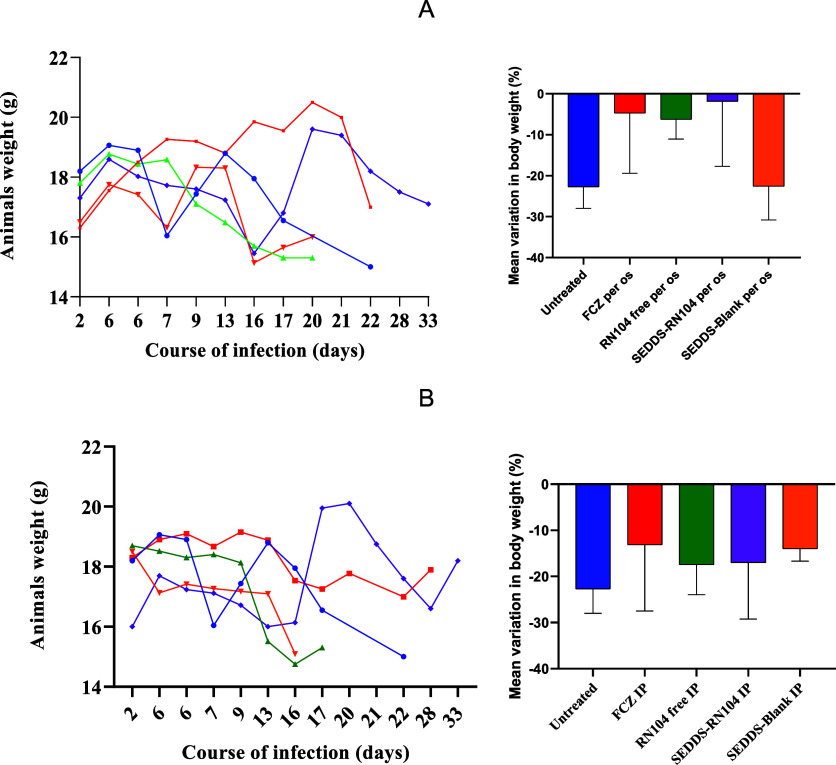
Weight variation of animals in each experimental group treated per os (A) and intraperitoneally (B) over the course of infection (days).

When assessing the intraperitoneal route (Figure 2B), no significant differences (*p* < 0.05) were observed between the groups treated with FCZ and SEDDS-RN104 (χ^2^ = 0.1013; *p* = 0.7503), nor when comparing free RN104 with SEDDS-RN104 (χ^2^ = 0.0450; *p* = 0.9463). The blank formulation administered intraperitoneally also did not cause toxic effects, as there was no statistical difference in the survival curve between SEDDS-Blank and the untreated group (χ^2^ = 0.0450; *p* = 0.9463). When evaluating the mean change in body weight, a significant difference was noted between the noninfected and treated groups. However, the various treatments had no significant differences (Figure 3B).

The survival curve results for SEDDS-RN104 administered per os may be related to its favorable pharmacokinetic profile. This formulation significantly enhanced the drug parameters, including a prolonged half-life (*T*_1/2_), reduced clearance, and substantial increase in the maximum concentration (*C*_max_). Remarkably, the bioavailability of SEDDS-RN104 was around 21 times higher compared to free RN104.^15^ By overcoming the inherent pharmacokinetic limitations of RN104,^13^ this formulation has the potential to significantly improve therapeutic efficacy.

### Fungal Burden Is Reduced in Mice Treated with SEDDS-RN104

Treated groups exhibited reduced pulmonary and cerebral fungal burdens compared to the untreated control (Figure 4). In cerebral tissue (Figure 4A), the untreated group presented a mean fungal load of 1.46 × 10^5^ CFU/g. In contrast, animals treated with FCZ demonstrated a significant reduction with a mean load of 2.70 × 10^4^ CFU/g. Similarly, animals treated with free RN104 and SEDDS-RN104 showed mean values of 5.29 × 10^4^ and 1.45 × 10^4^ CFU/g, respectively. Statistical analysis confirmed a significant reduction in fungal load in all treated groups compared to the untreated animals (*p* = 0.0065). While no significant difference was found among the RN104-treated groups due to limited fungal recovery, RN104 showed promise in reducing cerebral fungal burden. Fungi were recovered from only one animal in each RN104-treated group (*n* = 6), compared to three animals in the FCZ-treated group (*n* = 6).

**Figure 4:**
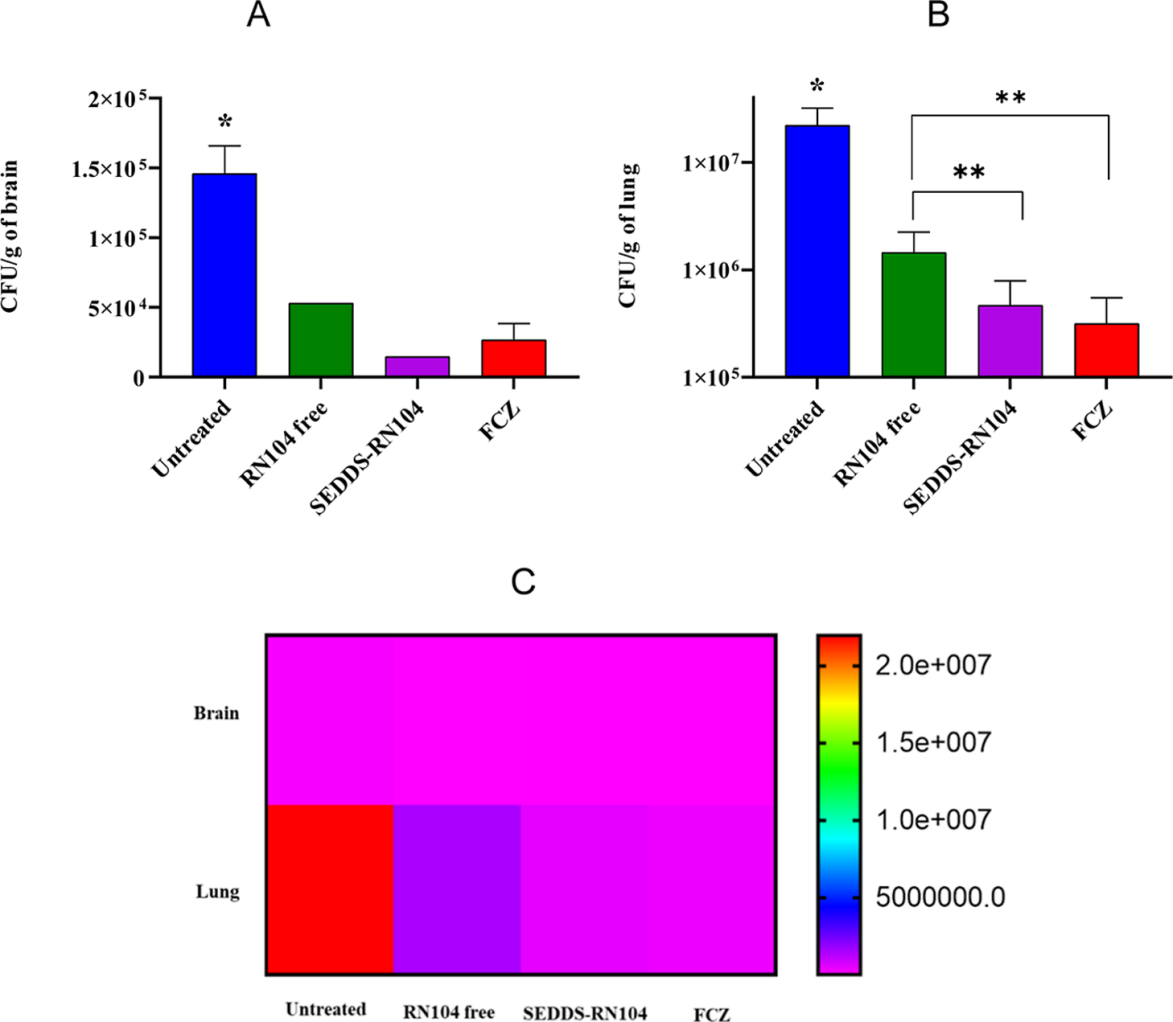
Fungal load (CFU/g) in the brain (A), lung (B), 10 days post-intratracheal infection with 1 × 10^5^ *C. neoformans* H99 cells and daily treatment per os. Data are presented as the mean ± standard deviation. *p < 0.05 compared to other groups; **p < 0.05 between treatment groups. FCZ: fluconazole and (C) heatmap of fungal burden in brain and lung tissues.

As shown in Figure 4B, the fungal load in the lung was significantly lower in all treated groups compared to the untreated group (*p* < 0.0001). The mean CFU/g was 1.76 × 10^7^ in untreated animals and 1.62 × 10^6^, 5.56 × 10^5^, and 4.77 × 10^5^ CFU/g for free RN104, FCZ, and SEDDS-RN104, respectively. While SEDDS-RN104 demonstrated superior efficacy to free RN104 in reducing pulmonary fungal burden (*p* < 0.05), SEDDS-RN104 exhibited comparable efficacy to FCZ (*p* = 0.9788), highlighting its potential as a therapeutic option for cryptococcosis.

Figure 4C is a heatmap that shows mean fungal burden (CFU/g of tissue) represented by a color gradient ranging from red (higher fungal load) to violet (lower fungal load). Data indicate a marked reduction in fungal burden in lung tissue for animals treated with SEDDS-RN104 and FCZ compared to the untreated control. Brain fungal burden remained consistently lower among all groups.

### Enhanced Pulmonary Histological Profile in Mice Treated with SEDDS-RN104

Pulmonary histological analysis (Figure 5) revealed diffuse and intense inflammatory infiltration, along with the destruction of the alveolar wall architecture in the untreated mice group. In contrast, the SEDDS-RN104-treated group exhibited fewer yeast cells (indicated by arrows), a mild inflammatory infiltrate, and preserved alveolar wall integrity when compared to untreated and other treated groups (*p* < 0.0001). Among the treatments, the SEDDS-RN104 group showed the least inflammation, with scores significantly lower than those of the FCZ group (*p* = 0.0179, Figure 5A).

**Figure 5:**
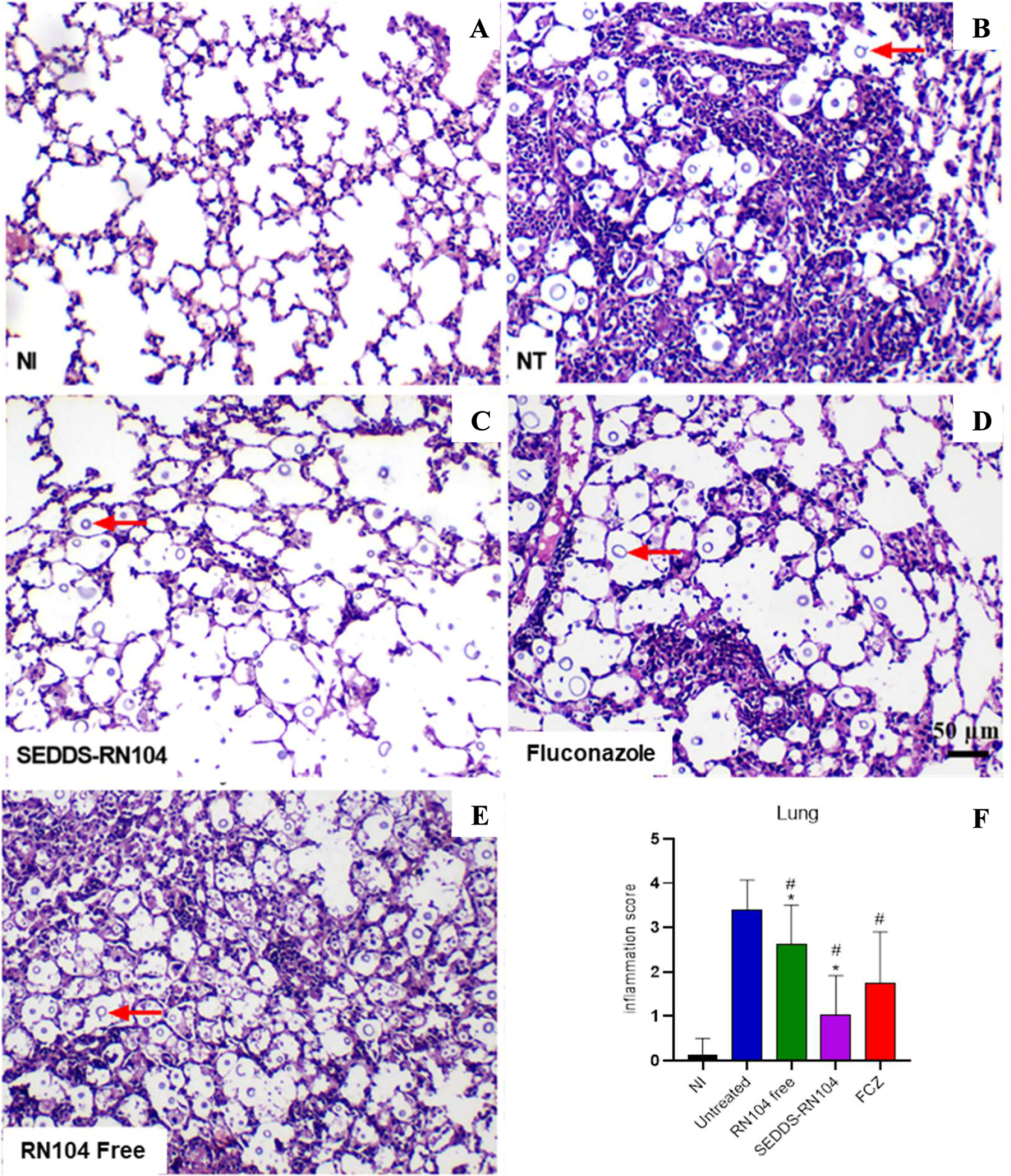
Representative histological images of lung tissue 10 days postinfection, illustrating the effects of different treatments per os (A–E) and the analysis of the inflammation score (F). Red arrows indicate yeast cells in the alveoli. Magnification: 100× scale bars: 50 μm. NI: not infected; NT: untreated; FCZ: fluconazole. #*p* < 0.05 compared to the untreated group; *p* < 0.05 compared to FCZ.

This study provides strong evidence that SEDDS-RN104 effectively reduces fungal burden in a murine model of cryptococcosis with efficacy comparable to FCZ in the lungs and promising results in the brain. Additionally, its ability to mitigate pulmonary inflammation represents a potential clinical advantage. These findings highlight the value of nanotechnology-based drug delivery systems in overcoming pharmacokinetic limitations and improving therapeutic outcomes for fungal infections. While recognizing that further validation is essential to clinical translation.

## Conclusions

This study demonstrated that SEDDS-RN104 significantly improved the antifungal efficacy of the compound in a murine model of cryptococcosis. The pharmacokinetic enhancements achieved with the formulation translated into superior therapeutic outcomes, including prolonged survival, a marked reduction in fungal burden, especially in the lungs, and decreased pulmonary inflammation. No signs of toxicity were observed during the study, supporting the safety profile. Given its efficacy is comparable to FCZ and the advantages of oral administration, SEDDS-RN104 stands out as a promising nanotechnology-based alternative for the treatment of cryptococcosis. Further studies are needed to validate its clinical translatability.
